# Progress in Surgery

**Published:** 1894-10-27

**Authors:** 


### Progress in Surgery.

### SURGERY OF THE ŒSOPHAGUS AND STOMACH.

**The Operative Treatment of Gastric Ulcer associated with Perforation.**—Mr. Gould states[1] that the symptoms are apt to be misleading and are no sure guide to the extent of the disease. Perforation occurs in 25 per cent. In 85 per cent. the perforation was on the anterior aspect of the organ opening into the peritoneal cavity. Death is occasionally due to shock, but generally occurs from peritonitis—the acute symptoms usually lasting twenty-four hours. Primary shock must be carefully distinguished from collapse. For its treatment little can be done beyond the administration of morphia and the application of warmth. The chief duty of the surgeon is to prevent or arrest the peritonitis. Too much stress has been laid on suturing the rent in the stomach, and too little on cleansing the peritoneum. The following measures should be adopted: (1) simple washing out of the abdominal cavity with salt solution or boiled water; (2) suture of the ulcer; and (3) where that is impossible, suturing the stomach to the abdominal parietes. Gould attributes no particular advantage to paring or excising the ulcer. The best incision, he thinks, is the mid-line, and the fluid used for flushing should be hot, as it then acts as a powerful restorative. R. Maclaren[2] has had three ceses, all fatal. He prefers an incision in the left linear semilunaris. R. Morison[3] has also operated on one case which ended fatally. Thomas H. Morse[4] has operated successfully on a case. No food was given by the mouth for sixty hours, and the patient was well at the end of three weeks. W. J. Maurice[5] operated on a case which died eight days afterwards. Hastings Gifford[6] has had a successful case. He excised the edge of the ulcer and sutured the walls with silkworm gut. A glass tube was placed in the neighbourhood of the ulcer and removed on the second day. Mr. Morse reports[7] a case which recovered, and Mr. Herbert Page[8] two others which were unsuccessful.

**Gastrostomy for Dilatation of Œsophagus.**—Kendal Franks has collected[9] twenty-one cases of non-malignant œsophageal stricture treated by gastrostomy and retrograde dilatation. All of these recovered except one case, in which the result is unknown. He does not consider operation in one stage (method of Loreta) to be admissible in cases of extensive injury to the œsophagus, caused by swallowing a strong corrosive. In such cases the operation *à deux temps* has an established and unassailable position in surgery. Tietze also draws attention[10] to the value of gastrostomy in these cases in (1) finding out the site of and treating the stricture, and (2) maintaining the nutrition of the patient. For these temporary gastric fistula Witzel's method is recommended. The author concludes that (1) gastrostomy should be done in severe cases more often than it has been in the past; (2) continuous dilatation with a drainage tube is more rapid and less dangerous than with bougies; and (3) combined gastrostomy and œsophagotomy may lead to success in some cases.

Witzel's method of performing gastrostomy seems to preclude all leakage of food through the abnormal opening. W. Meyer has found that seven months after operation no regurgitation took place, although with the lapse of time the external and internal openings were nearly opposite each other. F. T. Chavasse points out[11] the necessity in these cases of securely anchoring the indiarubber tube to the stomach wall by some unabsorbable sutures; for if catgut is employed, as in a case of his, the tube is apt to become displaced and its reintroduction difficult.

**Resection for Pyloric Carcinoma** is indicated—according to Zawadzki and Solman[12]—only when the tumour is small, well defined, moveable, and with no adhesions to adjacent organs, when there are no metastases, and when the cachexia is due to the pyloric obstruction rather than to the malignant disease itself. They draw attention to the degrees of dilatation of the stomach. If this is very great the food could hardly get through the new opening after the removal of the pylorus, hence a gastro-enterostomy at the lowest point should be performed. They narrate a case in which, after pylorectomy, the duodenum was sewn into the stomach and six lymphatic glands were removed. Nine months after operation the patient was doing well, but there was still no free hydrochloric acid present in the secretions of the stomach. A successful case is also reported[13] by Dr. Quenn, who made an anastomosis between the anterior wall of the stomach and duodenum by means of Murphy's button, and closed the wounds in the stomach and duodenum separately. The patient was well 65 days after operation. Dr. Tuffier also reports[14] a successful case—well eight months after; and Rosenheim has recently reported[15] three cases on whom he had performed operations for cancer of the pylorus with good result. Of these, one was known to be well three and a half years, and another three months after operation. It is known that an epigastric tumour is in the region of the pylorus, whenever, after artificial dilatation of the stomach with air or carbonic acid, the neoplasm is found at the upper right angle of the distended viscus. This sign, however, is not pathognomonic.

**Inflammatory Lesions Simulating Cancer of the Stomach** are sometimes greatly benefited by an operation, which is made merely exploratory, or of a more radical nature, according to the conditions found to exist. Professor Terrier narrates[16] such a case in a woman exhibiting all the characteristics of cancer, and coinciding with the existence of a perfectly appreciable epigastric tumour. On opening the abdomen he found extensive and intimate adhesions between the lesser curvature of the stomach and the left lobe of the liver, and lastly, some adhesions of the omentum to the upper part of the stomach. The adhesions were severed, and fragments of tissue removed for histological examination showed the lesion to be of a simple inflammatory nature. The patient rapidly recovered from the effect of the operation, and although for some months she continued to have trouble with her stomach, the disturbances gradually subsided. A year after the operation she was in excellent health, had gained in weight, and the tumour in the epigastric region had entirely disappeared.

[1] Lancet, Aug. 14th, 1894, p. 291. [2] Lancet, Aug. 14th, 1894, p. 292. [3] Ibidem. [4] Lancet, Mar. 17th, 1894, p. 671. [5] Lancet, June 2nd, 1894, p. 1373. [6] Lancet, June 2nd, 1894, p. 1369. [7] Lancet, Mar. 17th, 1894. [8] Lancet, Mar. 24th, 1894, p. 733. [9] Annals of Surgery, April 1894, p. 385. [10] Brit. Med. Jour., May 26th, 1894. [11] Lancet, April 28th, 1894, p. 1070. [12] Deut. Med. Woch., Feb. 22nd, 1894, and Brit. Med. Jour., Mar. 24th, 1894. [13] Med. Week, July 13th, 1894, p. 32. [14] Ibidem, p. 329. [15] Ibidem. July 6th, 1894, p. 321. [16] Ibidem, May 25th, 1894, p. 245.

